# Observations on Certain Parts of the Animal Economy, Inclusive of Several Papers from the Philosophical Transactions, &c.

**Published:** 1839-04

**Authors:** 


### PART SECOND.

### Bibliographical Notices.

Art. I.—*Observations on certain Parts of the Animal Economy, inclusive of several Papers from the Philosophical Transactions, &c.* By John Hunter, f.r.s. *With Notes, by* Richard Owen, f.r.s. &c. (*Being* Vol. IV. *of* Palmer's *Edition of the Works of* Hunter.)—*London*, 1837. 8vo. pp. 506.

We have already more than once noticed Mr. Palmer's edition of the works of John Hunter, and we shall probably refer to it on many future occasions. It is unquestionably the most valuable medical publication that has appeared since the commencement of our labours, and must claim a place in the library of every scientific member of our profession.

The volume to which we would now call the attention of our readers is, in some respects, the most interesting of the four. In it we see collected, for the first time, from various publications, those invaluable memoirs on subjects of natural history and physiology, which are not only full to overflowing of curious and important facts, but moreover contain the germs of many discoveries worked out by subsequent enquirers, as Bell, Magendie, Mayo, and others. The lapse of half a century since the publication of most of them has only tended to stamp more deeply their character for originality, fidelity, and wisdom. To speak of the whole works of John Hunter in other terms than of the highest commendation, or to refuse to acknowledge the depth, acuteness, and comprehensiveness of his genius, and assign him the first rank among physiologists,—now that the age of jealous rivalry is past,—would not be more unjust to his reputation than derogatory to the honour of our profession and country.

Our object in the present brief notice is chiefly to call the attention of the profession to the work itself, without any attempt at analysis or criticism : but we cannot refer to its contents without pointing out one or two of the numerous examples which it affords of facts, views, and opinions overlooked by Hunter's immediate successors, and promulgated, with more or less pretensions to novelty, by subsequent enquirers. We must also express, in a few words, our great satisfaction at the manner in which the task of preparing this volume has been executed by Professor Owen; than whom, perhaps, few men have evinced a more truly Hunterian spirit in prosecuting Hunter's favorite studies of natural history and physiology. He has indeed performed the laborious and self-imposed duty of annotating the original text in a most masterly manner, and with that fidelity and care which the importance of the object demanded. On every subject the information is brought up in the notes to the level of the present time; so that those who possess the "Animal Economy" in its new form will be able immediately to judge of the advance which science has made since the time of the author, and will perceive how vast a foundation was laid in these papers for after-discoveries. Mr. Owen has carefully pointed out many facts and principles originally established by Hunter, which have been more fully worked out and given to the world as new by succeeding physiologists. Thus, on the subject of *animal heat*, the principle that the adult animal has a higher temperature than the very young one, and that, after the animal has acquired the perfect or adult condition, it soon begins again to lose its power of generating heat, and approaches to the condition of the young animal, was first laid down by John Hunter, in his papers on this subject; although since generally attributed to Dr. Edwards, chiefly because it has been worked out by him. The most important facts elucidated by Dr. Edwards are clearly stated in the following extract:

"This variety (in the power of producing heat) not only takes place in animals of different orders, but in some degree in the same animal at different ages, even according to the different age of the parts in the same animal: a young animal requires more warmth than one full grown; and, although an animal is equally old in all its original parts, yet there are often new ones formed in consequence of diseases: and we find that these new or young parts in animals are not so able to support life as the old, at least for some time; but as animals are of different ages, and the same animal is always growing older, and of course more and more perfect, they then become more capable of generating heat than when they are younger. This, however, has its limitations; for, after a certain period, they again lose this power, and therefore require a less strongly conducting medium or warm atmosphere." (p. 134.)

Again, the facts stated by Dr. Davy, in his paper in the Philosophical Transactions for 1814, respecting the difference of temperature of different parts of the body in health, proving that those parts have the lowest amount which are farthest removed from the centre of circulation, appears to have been well understood by Hunter, as may be gathered from his observations on the temperature of different parts of the body, (pp. 140-2,) and more directly from the following remark:

"From experiments on mice and upon the dog, it plainly appears that every part of an animal is not of the same degree of heat; and hence we may reasonably infer that the heat of the vital parts of man is greater than either the mouth, rectum, or the urethra." (p. 146.)

The distinction which has been drawn by Dr. Marshall Hall, in his paper on *Hybernation*, in the Philosophical Transactions for 1832, between torpidity and sleep was equally well known to Hunter, as is remarked by Mr. Owen (*note*, p. 144;) and it is a somewhat curious circumstance that Dr. Hall has quoted other parts of the very paper in which Hunter points out the distinction, but has overlooked this, and also the statement at p. 144, that "beyond this point" (i. e. the degree of cold which acts as a sedative and produces sleep,) "cold seems to act as a stimulant, and rouses the animal powers to action for self-preservation." Dr. Hall also quotes the experiments of Hunter on the dormouse (Nos. xii. to xvi.) in illustration of this, as his own opinion respecting the operation of cold; but fails to notice the fact of this very opinion having previously been advanced, as we have shown, by Hunter.

On the subject of *Digestion*, Hunter's views have been proved to be equally correct with those on animal heat. The facts recorded in his celebrated paper "On the Stomach itself being digested after Death," have been fully confirmed by many of our most distinguished pathologists, as Baillie, Wardrop, Carswell, and others, both by observations made after death on the human subject, and (by Dr. Carswell) by experiments on rabbits, as noticed by us in a former Number of this Journal. We have ourselves repeated the experiments of Dr. Carswell on rabbits, and with nearly similar results. Moreover Prout, Children, Prevost, Le Royer, and more recently Drs. Beaumont and Dunglison, have all confirmed Hunter's opinions respecting the acidity of the gastric juice.

What led the mind of John Hunter so far to surpass his contemporaries, and anticipate subsequent enquirers upon almost every subject, was his persevering and determined pursuit of facts obtained by careful observation, and the almost entire dependence of his opinions upon what was supplied by anatomy, and had actually been examined by himself. It was this mode of proceeding which led him almost instinctively to perceive the laws by which the different functions of the animal body are regulated, and the relative position which animals occupy in the scale of creation. We would hold up Hunter's mode of proceeding to all who are desirous of establishing a reputation for science upon a sure basis, as the only means by which it can be rendered permanent and great. As a proof of the correctness of this opinion, it may be remarked that, where Hunter failed to form correct views with regard to the nature and uses of parts, the defect arose from his not having studied facts with his usual degree of scrupulous attention. An instance of this kind may be seen in his opinion with regard to the use of the vesiculæ seminales. Hunter regarded these structures not as seminal reservoirs, as they had been considered by previous physiologists, but as glands secreting a peculiar mucus, (p. 33:) he, however, acknowledged that he was unable to ascertain their peculiar use. Now, had he examined these structures with his wonted care, and had compared them with similar structures in all other classes of animals, particularly in many of the invertebrated tribes, to which upon some other points he devoted so much attention, he would probably have arrived at a different opinion, and have considered their primary use to be that of receptacles for the semen, as they had previously been thought; and perhaps also, as recently suggested by Dr. Davy, (see our last Number,) and also by Hunter himself, as secreting a mucus to be mixed with the semen for the purposes of generation.

